# Critical Analysis of The Changes in CFM Resolution 2294/21 And Its Impacts on Assisted Human Reproduction

**DOI:** 10.5935/1518-0557.20220007

**Published:** 2022

**Authors:** Fabio Roberto Cabar, Matheus Abelo de Oliveira, André Newman Cordeiro Machado

**Affiliations:** 1 Faculdade de Medicina da Universidade de São Paulo - Departamento de Obstetrícia e Ginecologia. São Paulo, SP, Brazil; 1 Centro Universitário - Faculdade das Américas. São Paulo, SP, Brazil

**Keywords:** Assisted Reproduction Techniques, Bioethics, Infertility, Standards of Medical Practice

## Abstract

The Brazilian Federal Board of Medicine (CFM) issued resolution number 2294/21, which regulates human reproduction procedures in Brazil, bringing significant changes to clinical practice in assisted human reproduction, and it raised ethical, bioethical, and legal discussions between professionals and patients. This study aims to analyze these changes in different aspects, especially because some of them are controversial. Evidence-based knowledge resources were used to support the analyses of crucial points that were impacted by this change. A literature review was carried out to obtain information about guidelines and laws, as well as articles that contemplate ethical discussions on assisted reproduction. The search sites used were BVS, Pub Med, LILACS and Google Scholar. The keywords used were law, legislation, bioethics, reference guide and assisted human reproduction. Relevant official documents from the Brazilian State were also found and included in the survey. The new resolution regarding the use of assisted reproduction techniques brought important changes, with clinical implications for couples who wish to become pregnant, and there is a need for a broad discussion concerning these repercussions from clinical, ethical, bioethical, and legal points of view.

## INTRODUCTION

Infertility is a health problem with medical and psychological implications, affecting about 15% of couples who try to become pregnant worldwide (Agarwal *et al*., 2015). This can become a large obstacle to the harmony of a couple’s life, causing instability, divorce, and polygamy, which can easily become a social stigma (Vayena *et al*., 2002).

Since it is an issue that reaches beyond the biological sphere, infertility has been highlighted as a triggering factor for violence against women in many societies (Murthy *et al*., 2010), requiring critical and profound insights, allied to a multidisciplinary network concerning the search for an effective treatment for infertile couples.

In the last two decades, the use of assisted reproduction techniques has grown significantly, especially in developing countries, such as Brazil, and the official rules regarding bioethics also undergo constant changes. In this paper, we will discuss the main changes brought on by Brazilian Federal Board of Medicine (CFM) in resolution number 2294/21.

## DISCUSSION

This paper will discuss the most relevant aspects of this new resolution, and to simplify things, will present it under topics of relevance.

## THE USE OF ASSISTED REPRODUCTION TECHNIQUES BY HOMO-AFFECTIVE COUPLES AND TRANSGENDER PEOPLE

The new resolution reinforces the possibility of people and couples without a diagnosis of infertility to use assisted reproduction procedures, such as people who want “a single parent”, homo-affective couples and transgender people.

Social inclusion is necessary and brings about the debate of important aspects concerning clinical practice in assisted reproduction. It should be noted that the guarantee of reproductive rights for transgender people is a recent topic in Brazil, but it has an ethical and legal basis. The CFM resolution follows the recommendations of important international medical associations, such as the American Society for Reproductive Medicine and the European Society of Human Reproduction and Embryology. These associations reinforce the need to equalize access to fertility options between cisgender and trans patients, and to discuss fertility preservation options before the beginning of the patients’ gender transition. (Ethics Committee of the American Society for Reproductive Medicine, 2015a; De Wert *et al*., 2014).

Recent publications show that the desire to start a family among “trans” patients is numerically relevant. A Belgian study reported that 54% of transsexual men wanted to have children, and 37% of them would have considered the possibility of freezing gametes if the option had been presented (Wierckx *et al*., 2012). As for the need to preserve fertility, a German study showed that 76% of both trans men and women had reflected on fertility preservation options before starting the transition (Auer *et al*., 2018).

Other authors showed that the results of ovarian stimulation in trans patients are like the results obtained from cis gender patients, even after the initiation of hormonal treatment with testosterone (Leung *et al*., 2019). Therefore, for these patients, as well as for transsexual women, the constitutional right, and the tangible possibility of carrying out family planning are assured.

Physicians and reproductive care providers are responsible for understanding the specific needs of transsexual patients. A study that discusses cultural competence in fertility care for lesbian, gay, bisexual, transgender and queer people demonstrate that these populations face unique obstacles in fulfilling their reproductive desires, as they experience heteronormativity, social stigmas, and specific psychological complications (Kirubarajan *et al*., 2021).

The lack of information and adapting services for care of historically marginalized groups is a valid and pertinent concern for care providers and patients alike (Kirubarajan *et al*., 2021). The inclusion of transgender people in the CFM resolution sheds light on this discussion and foreshadows the importance of seeking professional and scientific updating to better serve these patients.

## GAMETE OR EMBRYO DONATION

The latest study on the IVF scenario in European countries showed that marital status and sexual orientation are often seen as limitations for assisted reproduction technologies (ART). However, 34 out of the 43 countries have legal age limits for being eligible for ART. Belgium, Kazakhstan, and Malta set a minimum age for women, but they do not have this limitation for men. The maximum female age is also a legal limit in 18 countries, ranging from 45 years in Denmark and Belgium to 51 in Bulgaria. In Austria, they have the ‘natural cycle available’, which is an undefined criterion for a maximum age. The maximum male age is legally fixed in Portugal (60 years), and it is a recommended one in Finland (60 years) and Sweden (56 years). According to Swiss regulations, “the potential parent must be able to live until the child turns 18. In France, where there is no definition of age limits, it is up to the centers to define their own concept of ‘normal reproductive age’ (Calhaz-Jorge *et al*., 2020).

The new CFM resolution brings some alterations concerned gamete donation, compared to the last resolution. For now, women can donate oocytes until 37 years of age (before, they could be an egg donor until 35 years of age), while men can donate sperm until they turn 45 (in the previous resolution they could be donors until 50 years of age). Maternal age is one of the main causes of infertility in women. The reduction of ovarian reserve and decreased gamete quality, especially the increased incidence of aneuploidies due to aging damage, are the principal reasons for this (Badalotti & Petracco 2018). Woman’s age at the time of ovarian stimulation and egg retrieval, and the number of oocytes stored are crucial parameters in the cost-effectiveness of the treatment (Doyle *et al*., 2016). A study that analyzed the ploidy of more than 1,300 oocytes revealed that the frequency of aneuploidy remains relatively stable between ages 20 to 35 years, ranging from 5.2 to 10%. However, between the ages of 35 and 40 years, there is a significant increase in oocyte aneuploidy that remains between 12.5 to 28% (Pellestor *et al*., 2003).

Another study analyzed more than 15,000 embryo biopsies, and revealed a dramatic increase in embryonic aneuploidy rates in women aged 35 to 37 years (Franasiak *et al*., 2014). The age-related increase in aneuploidy of oocytes and embryos is probably related to a shift in meiotic competence due to abnormal meiotic spindle formation (Volarcik *et al*., 1998; Battaglia *et al*., 1996; Grøndahl *et al*., 2017).

Despite the availability of the new assisted reproduction technologies, the probability of an ongoing pregnancy being successful is also compromised by age. The chance of implantation after 40 years decreases by more than two-thirds, reflecting low embryo quality (Ziebe *et al*., 2001). In addition, late pregnancy is also associated with an increased risk of premature rupture of membranes, low birth weight, intrauterine growth restriction, and increased perinatal complications (Odibo *et al*., 2006; Reddy *et al*., 2006). This analysis aims to clarify that with the increase in the age group allowed for gamete donation, there is also the same relevance increase in the need for an open dialogue between doctor and patient, to clarify the lower rates of ovarian competence and oocyte quality, which intensify with advancing age; therefore, a progressive decrease in the probability of treatment success.

Regarding this age-limit for men, the relationship between the quality of the male gametes and the age of the donor is different, given that for men the reproductive function is maintained throughout life (Pasqualotto *et al*., 2008). However, according to recent studies that analyzed the profile of gamete production and DNA damage in males with advanced age, the impairment rates in genetic baggage increases, as well as other semen aspects such as semen volume, viscosity and quantity of sperm produced. Furthermore, the rate of germ cell repairs with DNA flaws was reduced, causing higher likelihoods of abortion (Colasante *et al*., 2018).

Probably the most relevant change brought on by the new resolution was associated with gamete donation. Historically, egg donation could only happen altruistically, by women who were undergoing assisted reproduction treatment. Later, couples who were not economically able to carry out their own treatment were allowed to donate eggs (shared donation) and, more recently, it was decided that women who were not undergoing any treatment for human reproduction could be oocyte donors, as long as there was no economic gain associated with it. Mutual confidentiality between egg donors and recipients was assured in all cases. From the publication of the new CFM resolution, the donation of gametes by relatives of up to four degrees is allowed as long as there is no consanguinity. It brings some ethical and legal concerns. There are different regulations and laws concerning the practice of donating oocytes in different countries. The literature on gamete donation among family members has focused mostly on disclosures among parents (Gottlieb *et al*., 2000; Lycett *et al*., 2005; Golombok *et al*., 2006; Lalos *et al*., 2007; Daniels *et al*., 2009). From these changes in Brazilian rules, a space has been opened for the choice between anonymity or disclosure of the identity of donors and recipients. In fact, some couples want to know the characteristics of the donor since they can have control over the origin of gametes and knowledge of social and medical history. If the donor is a family member, the couple may have the feeling of genetic continuity, but this decision has some problems. Sometimes, patient-specific conditions require the physician to go beyond the goal of conception (Comitê de Psicologia da SBRH, 2006). It is believed that, with greater understanding of the problem, patients can be better prepared, but this can only be achieved when physicians and patients have a higher joint awareness of their individual responsibilities. The identification between donor and recipient among family members is intrinsic to the process, which is therefore inevitable, since voluntary oocyte donors are basically driven by an altruistic desire to help infertile couples (Purewal & van den Akker, 2009; Svanberg *et al*., 2012). Thus, possible problems are associated with the secrecy about open disclosure to the child, family, and friends. Few studies address the issue, and the existing literature focuses on disclosure to the children, although recent data confirm the general well-being of families and children raised through donor-assisted reproduction (Söderström-Anttila *et al*., 2016).

The parents use different reasoning methods, and they include strategies such as: (i) rights-based reasoning, such as resolving tensions between a child’s right to know the means of his/her conception and a parent’s right to choose privacy; (ii) principled reasoning, as the decision is guided by personal values, moral and ethical principles, such as the desire to be honest and open with the child; (iii) reasoning about the offspring’s well-being, if the revelation helps to polish self-identity; (iv) reasoning about the well-being of the family, such as the consequences on interpersonal family relationships and whether confidentiality is inherently harmful to the family relationship; (v) context-dependent reasoning, as the decision is made based on a hypothetical context, such as the effects of the child accidentally learning about his/her genetic origins and feeling cheated (Blyth *et al*., 2010; Hahn & Craft-Rosenberg, 2002; Hershberger *et al*., 2007; Shehab *et al*., 2008).

To date, few studies have explored the aspects involved in this kind of donation, and what impact the relationship between donors and recipients can have, and their disclosure decisions. (Khamsi *et al*., 1997; van Berkel *et al*., 2007; Weil *et al*., 1994; Winter & Daniluk, 2004; Yee *et al*., 2007). The main obstacles that impact this decision are: (i) some family members of the donor and/or recipient and/or social network will likely know about the donation, including the identity of the donor and/or recipient; (ii) the donor and recipient experience a family or social relationship prior to the donation, which is likely to continue in the future, and will include the child conceived by the donor and any children of the donor; and (iii) when a donation occurs between family members, a child conceived by the donor will share a family relationship as well as a genetic relationship with the donor and his/her children. Given these unique characteristics, such disclosure decisions also include whether to disclose the donor’s identity, and possibly his/her children’s. In this scenario, the ways of conception and family life after the donation are presented as an important key to be guided by precise regulations, which rules for possible conflicts of interest on the maintenance of confidentiality. Furthermore, since the donor-recipient relationship is likely to continue after the donation, the decision to disclose this information to the child may have repercussions for others, especially among the donor and the recipient families.

Therefore, the update of the norms fails to mention specific and important points in the donation process between family members. It is important to have a precise recognition of behaviors about the professional’s role in anticipating and discussing possible conflicts, guided by norms that clarify more precisely the extension of their role beyond the very objective of the conception. The normalized, non-arbitrary relationship between donors, recipients and children must be considered in this scenario, since family relationships and the decision of maintaining the confidentiality can be modified over the years (Kalampalikis *et al*., 2018).

## CRYOPRESERVATION OF GAMETES AND EMBRYOS

The new CFM norm determines that “the total number of embryos generated in the laboratory cannot exceed eight. Patients must be instructed to decide how many fresh embryos they want transferred. Viable surpluses will be cryopreserved.”

There is a lack of reasonable justification for the arbitrary limitation of a maximum of eight embryos generated in the laboratory, regardless of a woman’s age. This decision has important implications in clinical practice, ethics, and individual rights.

In the scope of individual rights guaranteed by the Brazilian Federal Constitution, the limitation of the number of generated embryos interferes with the autonomy of couples who need assisted reproduction techniques. Invading philosophical discussions of Law, autonomy, and freedom to decide are unified in the concept of dignity, which is listed in the Brazilian Federal Constitution (1988). Based on the principles of human dignity, the Federal Constitution guarantees the right to family planning to couples; this right is regulated by an ordinary Law (number 9,263/96) (Diário Oficial da União, 1996).

On the order hand, the assistance to conception must be understood as a complex entity of clinical practice in assisted reproduction, precisely because of the singularity of each case. Personalized attention to the needs of each patient is harmed if the physicians’ performance is limited, specially when the limitation of the number of embryos does not consider the reality of medical practice and disregards the fact that reproductive difficulties are distinct and increase with advancing women’s age.

Ovarian reserve, for instance, is one of the factors that directly influences the individual reproductive capacity of women and couples. A reduction in the quantity and/or quality of oocytes with age is expected. One must rely on the logic that women who seek assisted reproduction intend to prolong their reproductive capacity (Busso *et al*., 2018). Decreased ovarian reserve represents 23% of indications for treatment in infertile couples. In women with the best prognoses, the live birth rate per cycle does not exceed 50% and this rate decreases inversely proportional to age, reaching 11.7% in the age group between 41 and 42 years (CDC, 2014). The quality and quantity of captured oocytes and the quality of laboratory manipulation of possible future embryos are factors that may influence the results.

A study that analyzed 20,687 women, found that live birth rates after the first cycle of assisted reproduction dropped from 63.8% in women under 31 to 4.7% in women aged 40 and over. On the other hand, the rate of live births after the first cycle rises with the increase of oocytes retrieved (Zhu *et al*., 2018). A previous study, which analyzed data from 256,381 cycles of in vitro fertilization with fresh embryo transfer, had already shown the increase in live birth rates with the highest number of oocytes (Steward *et al*., 2014).

These facts are the main aspect of the criticism to the new CFM resolution; the limitation of the number of fertilized embryos will affect the treatment results of women over 40 years of age, especially when compared to younger women. Additionally, the changes brought on by this new resolution impact the couple’s financial life and violates the citizen’s individual and reproductive rights. In addition, it is necessary to point out the possible implications of these changes to the scientific investigation; cryopreserved embryos are used to generate knowledge and these restrictions can affect science in Brazil. The imposition of a limitation on embryo production and the need for judicial permission for the disposal of embryos have no apparent legal or scientific justifications, which allows people to believe that these changes are related to ideology. Regarding the need for judicial authorization for the disposal of cryopreserved embryos, the motivations and inevitable consequences of this change must be discussed. The bureaucratization of this process generates higher costs for patients and contributes to the overload of the judicial system.

## PREIMPLANTATIONAL GENETIC DIAGNOSIS OF EMBRYOS

The diagnosis of the genetic sex of embryos through assisted reproduction technologies permeates one of the main pillars of the bioethical discussion on human reproduction. The new CFM resolution determines that assisted reproduction techniques can be applied to select embryos with disease-causing genetic alterations. However, it is allowed to inform the genetic sex of the embryo only in cases of sex-related diseases or sex chromosome aneuploidies. In fact, this prohibition was already included in the current Code of Medical Ethics, but lacked clear control and enforcement mechanisms. Thus, the new standard sets the limits for the disclosure of this information. There are several studies that address this issue, reflecting its large population impact. These publications bring positive and negative criticisms about embryo selection, primarily in view of the gaps that this decision opens for the selection of embryos in an arbitrary way, for personal reasons.

Those who live in countries where it is possible to select the sex of embryos argue that this choice should even be allowed, as it is the right of couples to determine what the composition of their families should be (Steinbock *et al*., 2002; Macklin *et al*., 2010). On the other hand, among those who criticize this possibility, the main concerns are related to the future of the offspring, as parents who select the sex of their children can impose norms related to the gender of their children, which would be harmful for the development of these children, especially those of the female sex (Kalfoglou *et al*., 2010; Ethics Committee of the American Society for Reproductive Medicine, 2015b). A concern of the Ethics Committee of the American Society for Assisted Reproduction is that sex selection can lead to a gender imbalance and social instability, and it violates the ethical principle of justice, considering that only those who can bear the costs of the procedure will be able to choose the sex of embryos. However, the committee does not have a consensus on whether it is ethical or not for assisted reproduction clinics to allow sex selection for non-medical purposes, as arguments relating to patient autonomy and reproductive freedom have been offered in support of the practice.

## TEMPORARY CESSATION OF THE UTERUS

Surrogate pregnancy is a method of pregnancy widely used by women with uterine-related infertility, as well as by same-sex couples and single men who want to achieve paternity through the creation of an embryo with their sperm and oocytes from donors. Traditional surrogacy consists of artificial insemination of the surrogate mother’s gametes with the father’s sperm, making it a genetic paternity along with the intended one. Gestational surrogacy is defined as an arrangement in which an embryo from the intended parents or from a donated oocyte or sperm is transferred to the surrogate uterus; therefore, the embryo does not have any kind of genetic sharing with the pregnant woman. The main indications for treatment are congenital or acquired absence of a functioning uterus and serious medical conditions that can be fatal to the pregnant woman (Brinsden, 2003).

Of all the countries in Latin America, only Uruguay has specific legislation regarding surrogate uterus. The Brazilian Congress has not enacted any regulation on surrogate motherhood, also known as “Solidarity Belly” or “temporary donation of the uterus”. In Brazil, it is forbidden to receive financial reward to do it, based on the Federal Constitution that interprets this practice as a form of trafficking in human organs. Responding to the lack of legislation, the Federal Board of Medicine created guidelines for altruistic surrogacy, which has been in force since 2010, and comprises the only set of rules applicable in Brazil to differentiate this practice from commercial surrogates. With the new update on ethical standards, it became mandatory for women who participate in surrogate pregnancy to have gone through at least one pregnancy that resulted in a living offspring. The update, which proves to be paternalistic, does not determine the preferable age group, nor does it suggest that the surrogate mothers have had a previous pregnancy without complications, an important point regarding maternal mortality rates, as suggested by the American Society of Reproductive Medicine.

It is known that pregnancy involves risks, which could justify this determination of the need for a woman who will be a surrogate to already have a child. However, there are other medical and surgical procedures that involve risks to patients (such as plastic surgeries, altruistic egg donation, etc.) and which, even so, can be performed by women if there is consent after proper information on the risks involved.

The lack of a legislation about that subject is a true concern, given that criminal law has been facing unregulated situations, such as the possibility of the affection between the temporary mother and the child generated during pregnancy, giving up maternity of both the intended mother and the pregnant mother in case of discovery of a malformation or multiple unwanted pregnancy and assistance in cost of the entire gestational process, and possible obstetric complications. In a UK study (Jadva *et al*., 2003) including 34 surrogate mothers, 35% initially had some difficulty in delivering the child. A year later, 6% still reported some negative feelings related to the resignation. Although problems of resignation sometimes occurred, a systematic review of studies on the subject revealed that most surrogates are within the normal range in personality tests (Söderström-Anttila *et al*., 2016). A retrospective cohort study collected data from 333 consecutive pregnancy cycles between 1998 and 2012, and revealed that the overall rate of maternal complications was only 9.8% (13/133), and an overall rate of fetal anomalies of 1.8% of babies born. The relatively low rates in this study were linked to a good obstetric history of all pregnant women and based on screening of oocyte donors.

The Brazilian Civil Law regulates the issue of paternity or maternity, in case of homologous or heterologous artificial insemination, in the presence of marriage or stable union. However, such legislation is shallow regarding reproduction if marriage or coexistence is absent, in cases of same-sex couples, or even about the use of replacement pregnancy. Brazilian standards do not guarantee stability between the parties involved. Legal enforcement of behavior is not possible due to the lack of laws. Therefore, prior counseling should raise all the points detailed above, including a detailed analysis of psychological status. Stability is currently only guaranteed through a mutual agreement between the parties after discussion of all foreseeable events.

## CONCLUSIONS

The new CFM resolution regarding the use of assisted reproduction techniques brought on important changes, with clinical implications for couples who wish to become pregnant, and there is a need for a broad discussion of these repercussions from a clinical, ethical, bioethical, and legal points of view.
